# The influence of Myalgic Encephalomyelitis/Chronic Fatigue Syndrome (ME/CFS) family history on patients with ME/CFS

**DOI:** 10.37349/emed.2024.00215

**Published:** 2024-04-11

**Authors:** Leonard A. Jason, Sarah Ngonmedje

**Affiliations:** Center for Community Research, DePaul University, Chicago, IL 60614, USA

**Keywords:** Myalgic Encephalomyelitis/Chronic Fatigue Syndrome, family history, gastrointestinal issues

## Abstract

**Aim:**

It is unclear if individuals with Myalgic Encephalomyelitis/Chronic Fatigue Syndrome (ME/CFS) with family histories of ME/CFS differ from those with ME/CFS without this family history. To explore this issue, quantitative data from patients with ME/CFS and controls were collected, and we examined those with and without family histories of ME/CFS.

**Methods:**

The samples included 400 patients with ME/CFS, and a non-ME/CFS chronic illness control group of 241 patients with multiple sclerosis (MS) and 173 with post-polio syndrome (PPS).

**Results:**

Confirming findings from prior studies, those with ME/CFS were more likely to have family members with ME/CFS than controls. We found family histories of ME/CFS were significantly higher (18%) among the ME/CFS group than the non-ME/CFS controls (3.9%). In addition, patients with ME/CFS who had family histories of ME/CFS were more likely to have gastrointestinal symptoms than those with ME/CFS without those family histories.

**Conclusions:**

Given the recent reports of gastrointestinal difficulties among those with ME/CFS, our findings might represent one predisposing factor for the emergence of ME/CFS.

## Introduction

Myalgic Encephalomyelitis/Chronic Fatigue Syndrome (ME/CFS) is a condition with multiple symptoms including post-exertional malaise, impaired memory/concentration, and unrefreshing sleep [1, 2]. Evidence exists that medical family illness factors may represent predisposing factors for ME/CFS [3–5]. Individuals with ME/CFS have a significantly higher number of blood relatives with diabetes (42.1%), lupus (7.0%), fibromyalgia (14.9%), and ME/CFS (5.3%) than non-blood relatives [6]. Also, Torres-Harding et al. [7] found that 50% of family members of patients with ME/CFS had endocrine/metabolic disorders compared to 28% among controls.

Endicott et al. [8] found patients with ME/CFS had mothers who died at an earlier age (mean = 59.5) compared to the two control groups (mean = 66.8 and 71.0) and these family members had a higher percentage of cancer (mean = 53%) compared to the control groups (mean = 33.1%, 31%). Smith et al. [5] found mothers of youth with ME/CFS had higher physical and mental fatigue, as well as higher levels of pain perception compared to healthy mothers. By analyzing the family history of patients with ME/CFS, medical problems appear to represent predisposing factors for the occurrence of ME/CFS.

In addition to those with ME/CFS having a greater probability of relatives having chronic health conditions, Walsh et al. [9] found that individuals with ME/CFS were more likely to have first-degree relatives with ME/CFS (i.e., rates of 4.3% compared to 0.5% among controls). Albright et al. [3] also found that relatives of people with ME/CFS were more likely to have ME/CFS, with first-degree relatives being the highest risk compared to other relatives. Finally, Rangel et al. [10] found that children with ME/CFS had parents with more ME/CFS-like diagnoses compared to parents of children with other chronic conditions.

In summary, the studies above suggest that people with ME/CFS are more likely to have a family member with ME/CFS, higher rates of cancer and autoimmune disorders, endocrine system dysregulation, mellitus, thyroid-related conditions, and Graves’ disease [3, 5–7, 9]. In our study, we first tried to replicate these findings of increased family history disorders among patients with ME/CFS. However, it remains unclear whether people with ME/CFS who have family members with ME/CFS have different symptoms than those without a family history. The current study explored whether those with a ME/CFS diagnosis with a family history of ME/CFS have symptoms that are different in type or severity than those with ME/CFS without a family history.

## Materials and methods

### Data collection and sampling method

The dataset for the current study was aggregated across a variety of populations. These data were from 400 patients with ME/CFS, and non-ME/CFS chronic illness control group of 241 patients with multiple sclerosis (MS) and 173 with post-polio syndrome (PPS) (the data sets are described in previous publications [11–13]). For the ME/CFS group, 72 had a family history of ME/CFS, and for the control groups, 8 patients with MS and 8 patients with PPS had a family history of ME/CFS. Participants were recruited through social media, email, and online support groups. A validated questionnaire [14] that was used originated from DePaul University and this questionnaire was administered to all those who participated in this study.

The DePaul University Institutional Review Board approved this study.

### Measure

The DePaul Symptom Questionnaire (DSQ-1). Participants across all datasets completed the DSQ-1 [14], a 54-item self-report measure of ME/CFS symptomatology, demographics, and medical, occupational, and social history. We selected this measure as it has been validated (see studies below). Participants were asked to rate the frequency of each symptom over the past six months on a 5-point Likert scale with 0 = none of the time, 1 = a little of the time, 2 = about half the time, 3 = most of the time, and 4 = all of the time. Likewise, participants were asked to rate the severity of each symptom over the past six months on a 5-point Likert scale with 0 = symptom not present, 1 = mild, 2 = moderate, 3 = severe, and 4 = very severe. The frequency scores and severity scores were standardized on a 100-point scale (each frequency and severity score from 0–4 was multiplied by 25, then added together, and then divided by 2). Furthermore, the frequency and severity scores for each symptom were averaged to create one composite score per symptom.

The DSQ-1 has demonstrated high test-retest reliability among persons with ME/CFS and controls [15], shown strong internal consistency [11], and yielded valid, clinically useful results [16]. DSQ-1 symptom domains include sleep, post-exertional malaise, neurocognitive, immune, neuroendocrine, pain, gastrointestinal, and orthostatic. The DSQ-1 is available in the shared library of Research Electronic Data Capture (REDCap) [17, 18]. The full questionnaire can be viewed here: https://redcap.is.depaul.edu/surveys/?s=tRxytSPVVw.

### Eligibility criteria

For the participants, we examined whether they had a family member with ME/CFS. Examples of family histories included answering “yes” to a question on the DSQ-1: “Have any of your family members been diagnosed with Chronic Fatigue Syndrome or Myalgic Encephalomyelitis”. If the participant answered yes, they were asked to provide more information regarding said family member(s) (i.e., age and relationship). In some cases, the family members wrote that they had a ME/CFS-like illness, and we counted them in the ME/CFS category. Ultimately, we categorized the patients into those who indicated that ME/CFS occurred in their family history *versus* those who did not have this family history.

### Statistical method

IBM SPSS Statistics version 26 was used to perform data analysis. For demographic variables, analysis of variance (ANOVA) and Bonferroni post-hoc tests were used for age, whereas chi-square analyses were used for the other binary variables. Chi-square was also used to evaluate the Family history of ME/CFS among the ME/CFS group versus the non-ME/CFS group. A one-way between-subjects ANOVA was conducted to compare the effect of a family history of ME/CFS on the DSQ-1 symptom domains: sleep, post-exertional malaise, neurocognitive, immune, neuroendocrine, pain, gastrointestinal, and orthostatic (a similar statistical test was used to examine specific symptoms within any domains that were significant).

## Results

The sociodemographic information on the four groups is provided in Table 1: those with ME/CFS and a family history of ME/CFS (*n =* 72), those with ME/CFS but no family history *(n =* 328), and control participants with a family history (*n* = 16) and control participants with no family history (*n* = 398). There were significant differences between the groups for age [*F* (3, 700) = 10.04, *p* < 0.01]. Bonferroni post-hoc tests indicated the two ME/CFS groups were significantly different from the control group without family history. There were significant overall differences between the groups for gender [*χ*^2^ (3) = 10.07, *p* < 0.05]. Examining two gender groups at a time, there was a significant difference between the ME/CFS with family history and the control group with family history [*χ*^2^ (1) = 4.74, *p* < 0.05)]. In addition, there was a significant difference between the ME/CFS with no family history group and the control group with no family history [*χ*^2^ (1) = 5.01, *p* < 0.05]. Collapsing racial groups into white *versus* other, there was an overall significant difference among racial groups [*χ*^2^ (3) = 9.97, *p* < 0.05]; there was a significant difference between ME/CFS with no family history group and the control group with no family history [*χ*^2^ (1) = 8.87, *p* < 0.05]. For marital status, we collapsed patients into three categories: married, separated/divorced/widowed, and never married, and there was a significant overall difference [*χ*^2^ (6) = 24.09, *p* < 0.01]. We found one marital status significant difference between the ME/CFS with no family history group and the controls with no family history [*χ*^2^ (2) = 18.12, *p* < 0.05]. Finally, when we categorized patients into four educational categories: less than high school, some high school, standard college, and graduate school, there was a significant difference [*χ*^2^ (9) = 18.24, *p* < 0.05]. For educational status, there was a significant difference between ME/CFS with no family history and controls with no family history [*χ*^2^ (3) = 9.85, *p* < 0.05].

As indicated in Table 2, family history of ME/CFS was significantly higher (18%) among the ME/CFS group than the non-ME/CFS group (3.9%), [*χ*^2^ (1) = 42.20, *p* < 0.05]. Because it would be expected that controls would have fewer symptoms than the ME/CFS groups, we focused our next comparisons on the domains of the DSQ-1 among ME/CFS samples (see Table 3). A DSQ-1 score incorporates both the presence and the intensity of a group of symptoms within a domain, like gastrointestinal symptoms. When comparing the effect of a family history of ME/CFS on the DSQ-1 symptom domains: sleep, post-exertional malaise, neurocognitive, immune, neuroendocrine, pain, gastrointestinal, and orthostatic, significance was only found for the gastrointestinal domain [*F* (1, 398) = 7.65, *p* < 0.01].

Due to the higher significance of the gastrointestinal symptom domain, we next examined specific symptoms within this domain listed in Table 4. We found a significant effect of family history on the gastrointestinal symptoms for abdomen/stomach pain [*F* (1, 398) = 11.04, *p* < 0.01] and irritable bowel problems [*F* (1, 398) = 4.17, *p* = 0.04]. These results suggest that having a family history of ME/CFS can have an influence on gastrointestinal issues such as abdomen/stomach pain and irritable bowel problems.

## Discussion

Our study has comparable outcomes to other studies in which patients with ME/CFS were more likely than controls to have family members with ME/CFS. Our second finding was unique and it involved those people with ME/CFS who have family members with ME/CFS, and they were more likely to have symptoms indicating gastrointestinal dysfunction than people with ME/CFS who do not have family members with ME/CFS. The higher rates of gastrointestinal problems among those patients with family backgrounds of ME/CFS suggest a mechanism by which there is a family correlation with ME/CFS. Perhaps it involves an actual pathogen the different family members contract or family members have an inherited condition affecting the gastrointestinal tract. Either way, it is possible this represents a possible predisposing factor for the development of ME/CFS.

The human gut microbiota is a powerful modulator of host immune responses and metabolism, and there is increasing evidence that chronic low-grade inflammation plays a significant role in the pathogenesis of chronic inflammatory-related diseases. Jason et al. [19] found that both before and at the onset of mononucleosis among college students, gastrointestinal symptoms were predictive of severe cases of ME/CFS six months after infection. Similarly, Jason and Dorri [20] found that gastrointestinal symptoms during the first few weeks of Coronavirus disease 2019 (COVID-19) were predictors of more serious consequences of COVID-19 about 6 months later. Johnson et al. [21] found that adults with ME/CFS report surprisingly high rates of youth gastrointestinal symptoms. Growing evidence from Guo et al. [22] and Xiong et al. [23] has found disruptions in the gastrointestinal microbiome among patients with ME/CFS. Furthermore, those changes are associated with an increase in pro-inflammatory species and a reduction in anti-inflammatory species [24]. These studies do suggest that not only are gastrointestinal problems serious among patients with ME/CFS, but that there might be an increased risk of having such problems among those with family histories of ME/CFS.

Family history can also be utilized to understand possible predisposing factors for those with ME/CFS. Youth and adolescent populations can also benefit from the use of family history by having symptoms screened early on which may even prevent the development of ME/CFS in adulthood. Family history can be used to better understand ME/CFS etiology, development, and treatment.

There are several limitations of this study. Although the primary comparison in the study was patients with ME/CFS and their family members, the control comparison was limited by different sociodemographic variables. In addition, our study did not confirm the diagnosis of ME/CFS in family members, by specialist examination and use of established ME/CFS case definitions. Also, the majority of symptoms were not significant among those with family histories *versus* those without family histories of ME/CFS, so the lack of other significant findings does suggest that there might be other predisposing factors rather than just family history. In addition, environmental factors regarding chronic medical problems were not thoroughly questioned in this study. Thus, future studies expanding on environmental factors would be useful to identify other factors that could be warranted as potential influences on ME/CFS.

In conclusion, the literature does suggest an increased prevalence of ME/CFS in family members of those with ME/CFS. Our study replicates these findings and adds to this literature by finding higher gastrointestinal symptoms among people with ME/CFS who had family histories of ME/CFS. We suggest that the influence of a family history of ME/CFS may be connected with more gastrointestinal issues among those with ME/CFS. Shared exposures among people with ME/CFS and their similarly affected family members might explain why they developed ME/CFS. It is also possible that the index cases with ME/CFS might have comparable predisposing conditions from other family members with ME/CFS. Family history studies can help in better understanding predisposing factors toward ME/CFS onset and maintenance and might lead to a better understanding of this illness and the people affected by it.

## Figures and Tables

**Table 1.** Sociodemographic information

Demographic variables	ME/CFS Family history (*n* = 72)	ME/CFS No family history (*n* = 328)	Controls Family history (*n* = 16)	Controls No family history (*n* = 398)
Age [years, mean (SD)]	48.42 (12.31)	49.86 (13.64)	56.27 (14.14)	55.55 (15.52)
Gender *% (n)*				
Male	7.35 (5)	12.38 (40)	26.67 (4)	18.44 (71)
Female	92.65 (63)	87.62 (283)	73.33 (11)	81.00 (312)
Other	0.00 (0)	0.00 (0)	0.00 (0)	0.52 (2)
Not reported	5.88 (4)	1.52 (5)	6.25 (1)	3.27 (13)
Race % (*n*)				
Black/African American	0.00 (0)	0.00 (0)	0.00 (0)	3.29 (13)
White/Caucasian	94.37 (67)	97.24 (317)	100.00 (16)	91.90 (363)
Native American/Alaskan Native	0.00 (0)	0.00 (0)	0.00 (0)	0.76 (3)
Asian or Pacific Islander	0.00 (0)	0.92 (3)	0.00 (0)	1.27 (5)
Other	5.63 (4)	1.84 (6)	0.00 (0)	2.78 (11)
Not reported	1.39 (1)	0.61 (2)	0.00 (0)	0.75 (3)
Marital status % (*n*)				
Married	46.48 (33)	50.46 (165)	75.00 (12)	62.97 (250)
Separated	4.23 (3)	2.45 (8)	0.00 (0)	1.01 (4)
Widowed	2.82 (2)	2.14 (7)	12.50 (2)	6.55 (26)
Divorced	21.13 (15)	16.82 (55)	6.25 (1)	13.85 (55)
Never married	25.35 (18)	28.13 (92)	6.25 (1)	15.62 (62)
Not reported	1.39 (1)	0.30 (1)	0.00 (0)	0.25 (1)
Education level % (*n*)				
Less than high school	2.78 (2)	2.45 (8)	0.00 (0)	1.01 (4)
High school/GED	13.89 (10)	6.13 (20)	12.50 (2)	7.79 (31)
Standard college degree	41.67 (30)	51.22 (167)	56.25 (9)	60.05 (239)
Graduate/professional degree	41.67 (30)	40.18 (131)	31.25 (5)	31.16 (124)
Not reported	0.00 (0)	0.61 (2)	0.00 (0)	0.00 (0)

SD: standard deviation; GED: General Educational Development

**Table 2.** Percent of patients with ME/CFS and controls with/without a family history of ME/CFS

Family history status	ME/CFS *(n* = 400)	Controls (*n* = 414)	Total	Significance level
	% (*n*)	% (*n*)		
Family history	18.0 (72)	3.9 (16)	88	< 0.05
No family history	82.0 (328)	96.1 (398)	726	

**Table 3.** Differences in DSQ-1 symptom domain scores among those with and without a ME/CFS family history

Symptom domains	ME/CFS Family history (*n* = 72)	ME/CFS No family history (*n* = 328)	Significance level
	mean (SD)	mean (SD)	
Sleep	62.58 (20.23)	61.06 (18.23)	0.53
PEM	73.36 (18.29)	72.13 (17.02)	0.58
Neurocognitive	58.25 (20.66)	60.12 (20.01)	0.47
Immune	36.62 (17.96)	34.15 (18.84)	0.31
Neuroendocrine	38.66 (18.75)	37.94 (20.79)	0.79
Pain	64.84 (26.31)	63.18 (26.67)	0.63
Gastrointestinal	52.31 (23.19)	43.37 (25.18)	< 0.01
Orthostatic	37.71 (16.79)	35.05 (19.20)	0.29

PEM: post-exertional malaise

**Table 4.** Differences in gastrointestinal symptom scores among those with and without an ME/CFS history

Symptom domain gastrointestinal	ME/CFS Family history (*n* = 72)	ME/CFS No family history (*n* = 326)	Significance level
	mean (SD)	mean (SD)	
Bloating	51.74 (27.62)	44.98 (28.35)	0.07
Abdomen/stomach pain	50.69 (24.90)	39.14 (27.10)	< 0.01
Irritable bowel problems	54.69 (30.86)	46.15 (32.41)	0.04

## Data Availability

The raw data supporting the conclusions of this manuscript will be made available by the authors, without undue reservation, to any qualified researcher.

## Abbreviations

DSQ-1: DePaul Symptom Questionnaire
ME/CFS: Myalgic Encephalomyelitis/Chronic Fatigue Syndrome
MS: multiple sclerosis
